# Primary orbital solitary fibrous tumor with an 18-year course of five recurrences and extensive sinonasal metastasis following eyeball enucleation: a case report

**DOI:** 10.3389/fmed.2026.1858538

**Published:** 2026-05-22

**Authors:** Jiayin Cai, Yijia Zhang, Jiabei Xiong, Xiaoxiao Luo, Yiyao Wang, Yongping Hu, Xiaolin Cao, Jianguang Zhong, Jian Li

**Affiliations:** 1 The Fourth School of Clinical Medicine, Zhejiang Chinese Medical University, Hangzhou First People’s Hospital, Hangzhou, Zhejiang, China; 2 Department of Ophthalmology, Affiliated Hangzhou First People’s Hospital, School of Medicine, Westlake University, Hangzhou, Zhejiang, China; 3 Department of Otolaryngology, Affiliated Hangzhou First People’s Hospital, School of Medicine, Westlake University, Hangzhou, Zhejiang, China

**Keywords:** disease management, enucleation of eyeball, orbital solitary fibrous tumor, sinonasal invasion, tumor recurrence

## Abstract

**Background:**

Orbital solitary fibrous tumor (OSFT) represents a rare mesenchymal neoplasm that typically follows an indolent clinical course. However, aggressive variants can exhibit a high propensity for local recurrence, extensive tissue invasion, and delayed metastasis, thereby posing significant challenges for clinical management. The long-term clinical trajectory of highly recurrent OSFT and the optimal multidisciplinary management strategies remain inadequately characterized. Our findings underscore the aggressive potential of recurrent OSFT and propose a comprehensive strategy for long-term surveillance and intervention.

**Case presentation:**

We report an exceptionally rare case of a female patient who experienced an 18-years protracted course of OSFT. Initially presenting at age 52 with proptosis and visual impairment, the patient ultimately underwent five surgical interventions due to repeated local recurrences. Despite repeated excisions, the disease progressed relentlessly, necessitating orbital exenteration during the fourth recurrence. Five years after exenteration, a fifth recurrence occurred with extensive paranasal sinus invasion, extending into the frontal, ethmoid, and maxillary sinuses. Histopathological and immunohistochemical analyses consistently confirmed the diagnosis of SFT, characterized by the hallmark by nuclear STAT6 positivity. Notably, the latest recurrence demonstrated increased cellular atypia, elevated mitotic activity, and a Ki-67 proliferation index of approximately 20%. The patient was subsequently treated with endoscopic tumor resection combined with postoperative adjuvant radiotherapy.

**Conclusion:**

Orbital solitary fibrous tumor can emerge as a highly aggressive neoplasm characterized by an insidious onset, a protracted recurrent course, and profound local invasiveness. This case strongly correlates repeated recurrences with the anatomical challenges of achieving complete initial resection. To prevent severe structural destruction and improve long-term outcomes, we advocate for early complete surgical excision, the judicious integration of targeted adjuvant radiotherapy for high-risk or locally invasive cases, and stringent, lifelong clinical and systemic imaging surveillance.

## 1 Introduction

**FIGURE 1:**
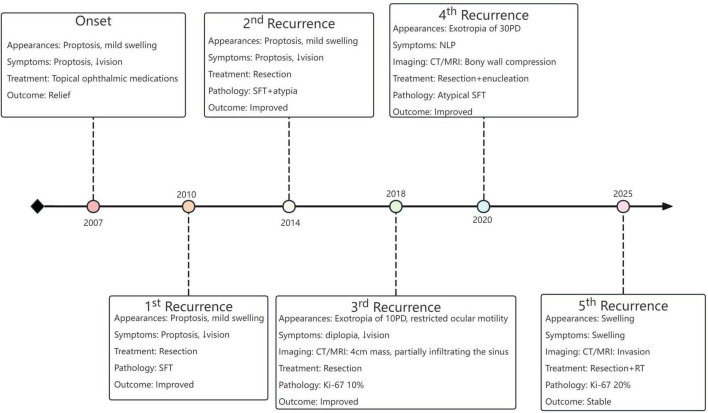
Clinical timeline of a patient with recurrent OSFT from 2007 to 2025, illustrating six disease courses including the primary presentation and five recurrences. Key appearances, symptoms, imaging findings, treatments, and pathological evolution are summarized. The tumor demonstrated progressive aggressiveness, with transition to atypical SFT and increasing Ki-67 index over time. SFT, solitary fibrous tumor; PD, prism diopters; NLP, no light perception; RT, radiotherapy.

Solitary fibrous tumor (SFT), a type of mesenchymal tumor defined by the pathognomonic NAB2-STAT6 gene fusion (1), was first reported in 1931 by Klemperer and Rabin (2). Pleural SFT represents the most commonly reported subtype (3). However, head and neck SFTs are relatively rare, accounting for approximately 5% of all cases (1), with the paranasal sinuses (about 30%) and orbit (about 25%) being the predominant involved (4). Accordingly, orbital solitary fibrous tumor (OSFT) accounts for only approximately 1.3% of all SFT. OSFT was first reported by Westra et al. (5), 63 years after the initial description of SFT. Typically, OSFTs exhibit benign biological behavior, characterized by slow growth and low metastatic potential (6). Following complete surgical excision, recurrence rates of OSFT is significantly reduced. Postoperative recurrence cases are rarely reported in the literature (7). Here, we present a rare case with an 18-years disease course, experiencing five recurrences. Following the fourth recurrence, orbital exenteration was performed to achieve maximally complete tumor resection. Nevertheless, a fifth recurrence subsequently occurred postoperatively, accompanied by extensive paranasal sinus metastases (Figure 1).

## 2 Case report

In 2007, a 52-years-old woman, with no relevant personal or family medical history, first presented with proptosis, visual impairment, ocular motility disturbance and epiphora in the left eye; the right eye was unremarkable even on subsequent examinations. Topical ophthalmic medications were adopted (details of the medication are not specified). The symptoms were relieved.

Three years later, in 2010, an orbital tumor resection was performed due to recurrence of symptoms mentioned above. Intraoperative pathological specimen confirmed a solitary fibrous tumor. After surgery, both proptosis and visual impairment in the left eye exhibited mild improvement.

Four years later, in 2014, the tumor recurred and was re-resected. The pathology was consistent with solitary fibrous tumor, with areas of cellular density and mild atypia, alongside cartilaginous tissue.

In 2018, the patient presented with a 6-months history of diplopia and visual impairment in the left eye, associated with orbital aching, metamorphopsia, proptosis, and occasional dizziness. On examination of left eye, the decimal visual acuity was 0.3; exotropia measured 10 prism diopters; restricted ocular motility of the left eye involving adduction, supraduction, and infraduction. Computed tomography (CT) demonstrated a heterogeneously enhancing soft tissue mass measuring 4.0 × 2.4 × 3.7 cm in the medial left orbit, causing compression of the superior wall of the left maxillary sinus and the medial wall of the ethmoid sinus. Magnetic resonance imaging (MRI) corroborated the CT findings and additionally indicated a vascular tumor. Surgical intervention was performed. Intraoperative findings revealed that the tumor was partially embedded within the maxillary sinus. Clinical symptoms were not alleviated. Immunohistochemical studies of frozen tissue sections of the excised tumor showed staining of the tumor cells for CD34, P53 and Ki67 (10%), with focal areas of hypercellularity and mitotic activity of 5 per high-power field.

**FIGURE 2:**
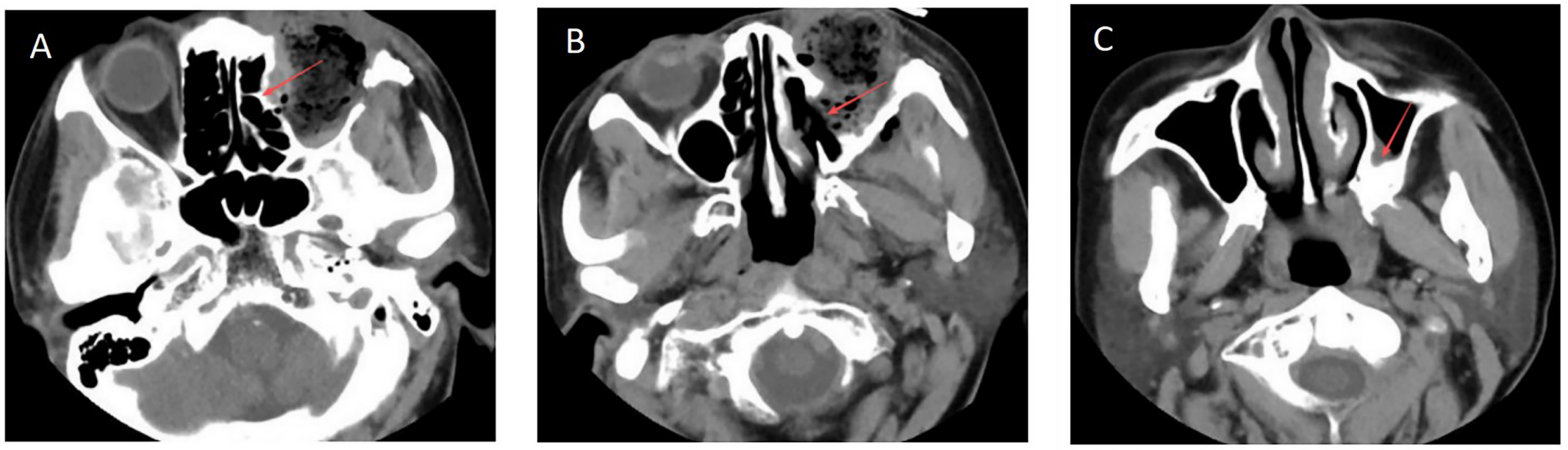
Postoperative orbital CT scan with three-dimensional reconstruction revealed localized inward depression of the medial wall of the left ethmoid sinus **(A)** and the superior wall of the left maxillary sinus **(B)**, accompanied by minimal exudative changes in the left maxillary sinus **(C)**.

In 2020, the patient presented with a 4-months history of no light perception (NLP) in the left eye accompanying the fourth tumor recurrence. Ophthalmic examination revealed intraocular pressure (IOP) of 17.6 mmHg in the right eye and 26.6 mmHg in the left eye, with left exotropia of approximately 30 prism diopters. A firm, tender orbital mass was palpable through the left eyelid along the nasal 180 degrees, with associated vascular congestion and dilation observed on the nasal bulbar conjunctiva. The patient underwent orbital tumor resection combined with left orbital exenteration and titanium plate implantation. Intraoperatively, a widely distributed, lobulated tumor was observed occupying the superior, nasal, and inferior aspects of the orbit. The mass exhibited a relatively intact capsule. Superiorly, it invaded the superior orbital fissure; inferiorly, it extended into the superior wall of the maxillary sinus. The tumor deeply involved the orbital apex and adhered tightly to the optic nerve. No obvious bony erosion was noted. Postoperative orbital CT scan with three-dimensional reconstruction (Figure 2) revealed localized inward depression of the medial wall of the left ethmoid sinus and the superior wall of the left maxillary sinus, accompanied by minimal exudative changes in the left maxillary sinus. Immunohistochemistry confirmed the recurrent tumor as atypical SFT, with immunopositivity for Ki-67 (10%), Caldesmon, STAT6 (nuclear), CD34, Bcl-2 and CD99.

**FIGURE 3:**
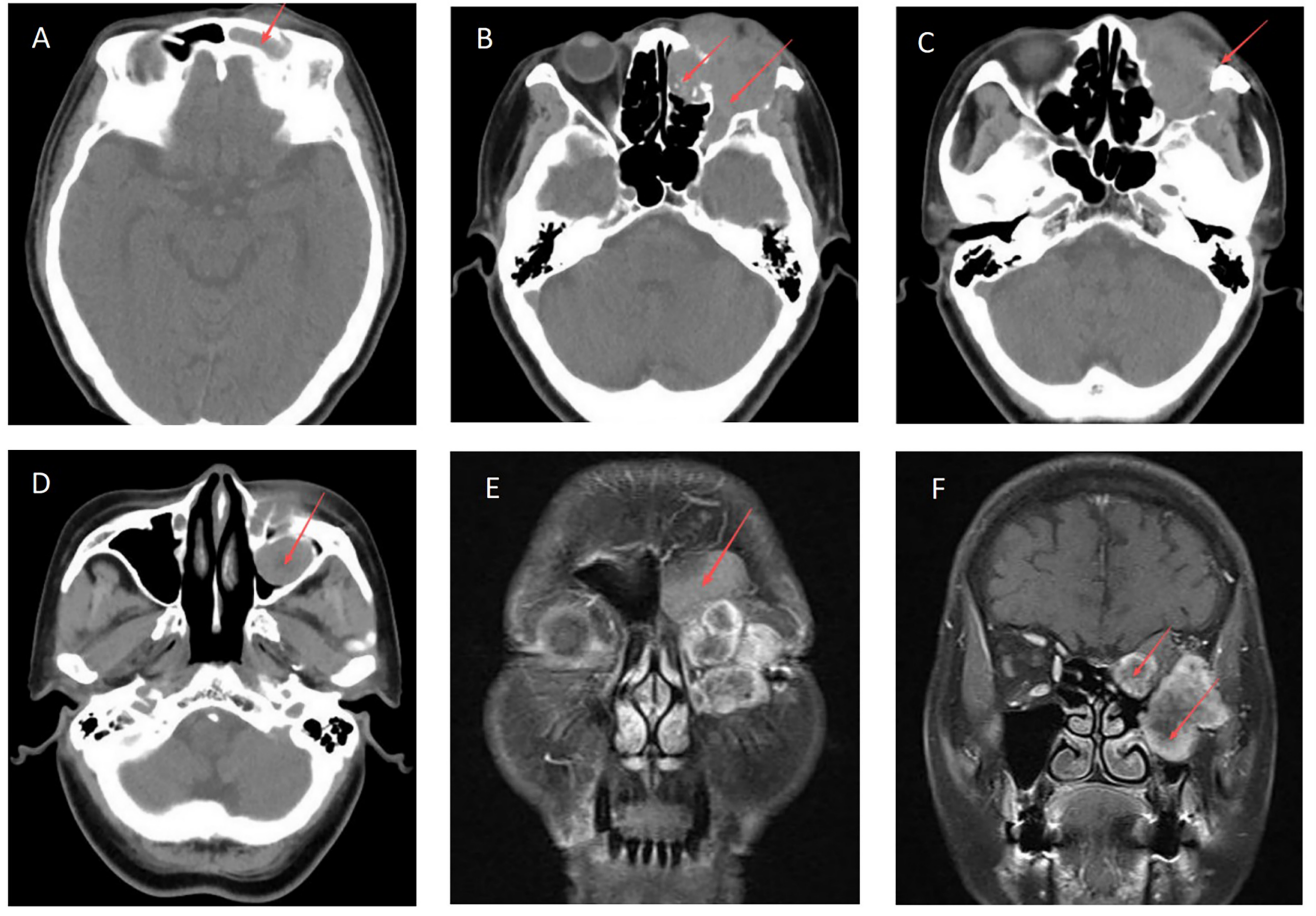
Computed tomography (CT) and MRI (T1) revealed an irregular soft tissue mass was observed within the left orbit, extending medially into the frontal **(A,E)** and ethmoid sinuses **(B,F)**, laterally to the zygomatic arch **(C)**, and inferiorly into the maxillary sinus **(D,F)**, with obliteration of the intraorbital fat planes **(B)**.

**FIGURE 4:**
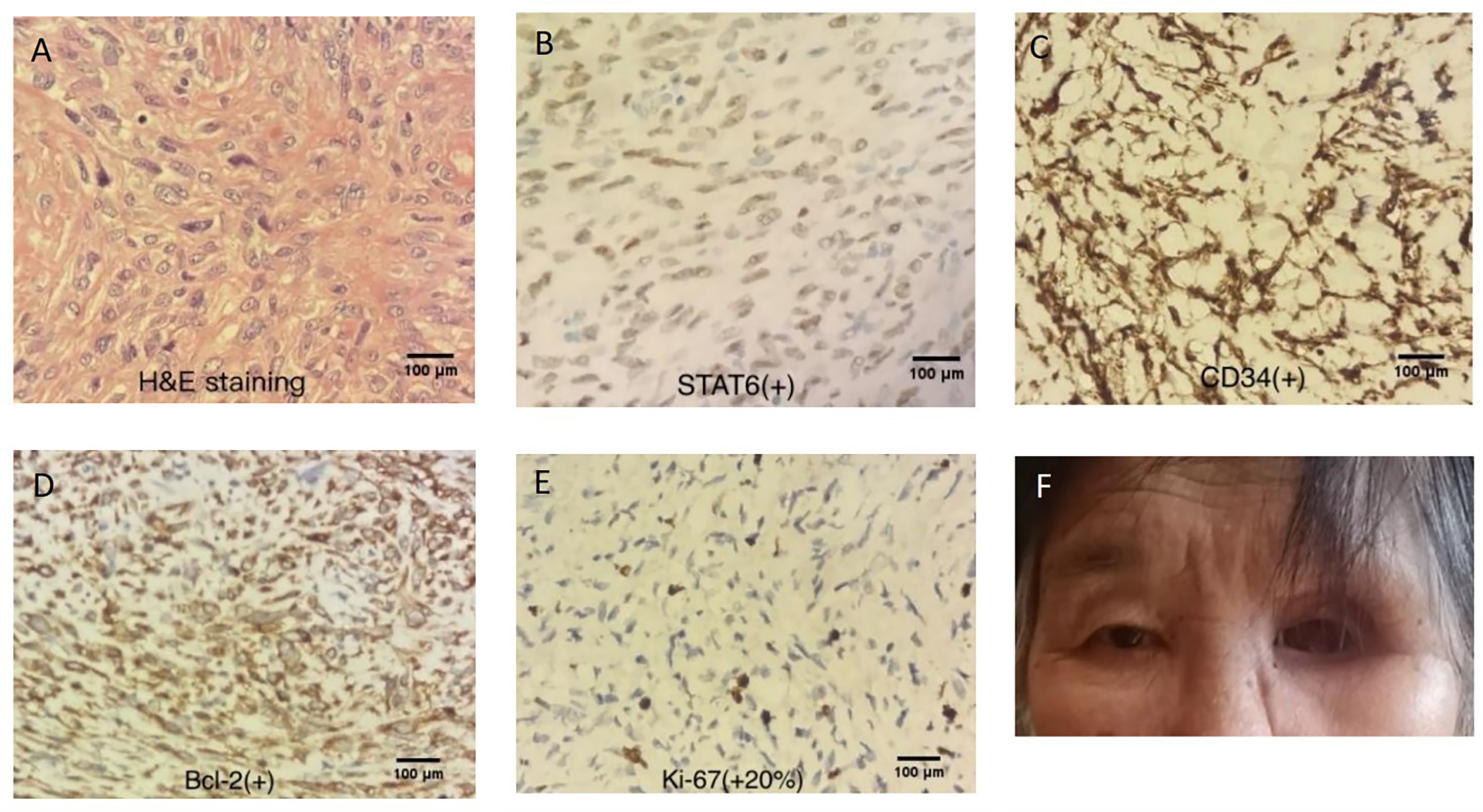
Intraoperative frozen section revealed a spindle cell mesenchymal tumor with increased cellular density and cytologic atypia, a mitotic count of >4 per 10 high-power fields (reaching 10 per HPF in hot spots), accompanied by necrosis and hemorrhage **(A)**. Immunohistochemical staining demonstrated positivity for STAT6 (nuclear) **(B)**, CD34 **(C)**, Bcl-2 **(D)** and CD99, with a Ki-67 proliferation index of approximately 20% **(E)**. Appearance of the patient after the fifth recurrence surgery and during ongoing radiotherapy **(F)**. Scalebar: 100 μm.

In 2025, when the patient was 70 years old, she returned to our hospital with clinical suspicion of tumor recurrence manifesting as left periorbital swelling, frontal facial discomfort, decreased epiphora, and occasional headache and dizziness. Physical examination revealed a palpable mass in the left orbital region involving the medial aspects of both the upper and lower eyelids, with suspected involvement of the lacrimal sac, resulting in inability to open the eyelid. Nasal examination revealed bilateral turbinate swelling with pale mucosa. A neoplasm was visible at the left frontal sinus ostium, and the medial orbital wall mass was observed protruding into the nasal cavity. CT and MRI (Figure 3) revealed recurrent tumor in the left orbit with extensive surrounding osteolytic destruction. An irregular soft tissue mass was observed within the left orbit, extending medially into the frontal and ethmoid sinuses, laterally to the zygomatic arch, and inferiorly into the maxillary sinus, with obliteration of the intraorbital fat planes. The patient underwent endoscopic resection of sinonasal lesions, endoscopic resection of intraorbital lesions, and reconstruction of the medial orbital wall. Intraoperative frozen section revealed a spindle cell mesenchymal tumor with increased cellular density and cytologic atypia, a mitotic count of >4 per 10 high-power fields (reaching 10 per HPF in hot spots), accompanied by necrosis and hemorrhage (Figure 4A). Immunohistochemical staining (Figures 4B–E) demonstrated positivity for STAT6 (nuclear), CD34, Bcl-2 and CD99, with a Ki-67 proliferation index of approximately 20%.

The patient underwent postoperative adjuvant radiotherapy, with the target volume covering the postoperative tumor bed and high-risk subclinical lesions. The prescribed doses were 69.96 Gy in 33 fractions (2.12 Gy per fraction) to the gross tumor volume of the postoperative bed (PGTVp) and 60.06 Gy in 33 fractions (1.82 Gy per fraction) to the planning target volume (PTV), delivered at 5 fractions per week. Radiotherapy is currently ongoing without significant discomfort. Postoperative follow-up examination revealed well-healed surgical cavities with superficial crusting and no evidence of new lesions (Figure 4F).

## 3 Discussion

Orbital solitary fibrous tumor is a rare spindle cell neoplasm of mesenchymal origin. Although it typically exhibits an indolent clinical course, it is associated with a high local recurrence rate, the potential for late distant metastasis, and the risk of malignant transformation. Imaging studies may reveal a space-occupying lesion; however, the findings are nonspecific and insufficient for a definitive diagnosis. Many orbital diseases share similar imaging features, and accurate differentiation relies on subtle radiologic signs and integrated clinical information, which requires considerable clinical experience and remains challenging for non-specialists. In this context, orbital MRI/CT may serve as an important initial evaluation tool in patients presenting with proptosis, strabismus, or ocular motility disturbances. When an orbital lesion is identified, timely referral to an orbital specialist is warranted for further diagnostic workup and management. Therefore, AI-assisted imaging diagnostic systems are expected to provide valuable clinical support in improving the accuracy and efficiency of differential diagnosis (8).

The definitive diagnosis of solitary fibrous tumor (SFT) is highly dependent on immunohistochemical profiling, with nuclear expression of STAT6 serving as a characteristic diagnostic hallmark (9). Although CD34, Bcl-2, and CD99 are frequently positive in SFT, their specificity is relatively low, and they are therefore considered ancillary markers (10). In the present case, postoperative pathological examinations following the fourth and fifth recurrences revealed a spindle cell neoplasm with an immunophenotype positive for STAT6, CD34, Bcl-2, and CD99, leading to a confirmed diagnosis of SFT. Histologically, SFT encompasses several subtypes, including classic, fat-forming, giant cell-rich, malignant, and dedifferentiated variants (11). Among these, dedifferentiated SFT is characterized by an abrupt transition from a low-grade SFT to a high-grade sarcoma and carries an extremely poor prognosis, with loss of CD34 expression occurring in approximately 0.85% of cases (12). Given the broad biological spectrum of SFT–ranging from indolent to overtly aggressive behavior–the World Health Organization (WHO) classification recommends a multiparameter risk stratification model that integrates patient age (>45 years), tumor size (>3.0 cm), mitotic count (>4/2 mm^2^), and the presence of necrosis to assess prognosis, rather than relying on a simple benign versus malignant dichotomy (13, 14). This model facilitates accurate identification of malignant SFTs with high potential for recurrence and metastasis.

At the time of the fifth recurrence, in addition to the primary orbital lesion, sinonasal involvement was identified, raising the differential diagnosis among true metastasis, multicentric origin, or direct contiguous invasion. The differential diagnosis primarily includes other tumors that may simultaneously involve the orbit and paranasal sinuses, such as other mesenchymal tumors, meningioma, and schwannoma: (1) Other mesenchymal tumors: Some tumors, particularly those with spindle cell morphology, may closely resemble SFT histologically, necessitating immunohistochemical differentiation. Nuclear expression of STAT6 demonstrates high specificity (96%) for SFT, with only 4% of other mesenchymal tumors showing positive nuclear staining (15). (2) Meningioma: Orbital meningioma may extend into the paranasal sinuses and exhibits significant radiological and anatomical overlap with SFT, both presenting as well-defined enhancing masses. Histologically, meningiomas may also display spindle cells. Immunohistochemistry is crucial for distinction: meningiomas typically express Somatostatin Receptor Type 2A (SSTR2A) and epithelial membrane antigen (EMA), whereas SFT demonstrates nuclear STAT6 positivity and CD34 expression, with SSTR2A and EMA usually being negative (16). (3) Schwannoma: Certain subtypes of SFT, particularly the paucicellular variant, may histologically mimic schwannoma (Antoni B areas), both being characterized by spindle cells embedded in a collagenous stroma. Conversely, some schwannomas, especially the cellular variant, may exhibit a hemangiopericytoma-like vascular pattern resembling that of SFT. Immunohistochemistry is essential for distinguishing SFT from schwannoma: SFT is characterized by nuclear STAT6 positivity and absence of S-100 and SOX10 expression, whereas schwannoma exhibits diffuse strong immunoreactivity for S-100 and SOX10, with STAT6 consistently negative (16). Immunohistochemical analysis, particularly STAT6 nuclear positivity, is essential for confirming the diagnosis of SFT and excluding other entities.

Complete surgical resection remains the first-line treatment for OSFT (17). However, achieving R0 resection poses significant challenges due to the tumor’s friable pseudocapsule, dense adhesions to surrounding tissues, and frequent infiltration into intraorbital adipose tissue, compounded by the narrow anatomic confines of the orbit and the presence of critical neurovascular structures (7). To address these difficulties, various optimized surgical strategies have emerged in recent years. Among them, preoperative embolization can effectively occlude tumor blood supply, significantly reduce intraoperative hemorrhage, and thereby improve the rate of complete tumor resection (18). Notably, when OSFT exhibits aggressive biological behavior, more extensive surgical resection may be warranted. It has been reported in the literature that orbital exenteration may be necessary for achieving local control in cases with aggressive features (19). Even in the setting of recurrent disease, surgical resection remains the preferred strategy (20).

Radiotherapy is considered an effective adjunctive treatment modality for OSFT (21). Combined surgical resection and radiotherapy have demonstrated favorable outcomes in select cases. However, long-term prognosis remains uncertain, with potential risks including rare but severe sarcomatous dedifferentiation following radiotherapy (22) and other potentially life-threatening complications (23). Therefore, the decision to administer adjuvant radiotherapy for low-grade tumors should be approached with considerable caution and guided by risk stratification results based on the WHO-recommended model. Upon review of the case, it was found that at the time of the fifth recurrence, the OSFT had extensively invaded the paranasal sinuses. In fact, during the third recurrence, the tumor had already shown signs of invading the bony wall of the maxillary sinus (the tumor was partially embedded within the maxillary sinus). Given the complexity of the orbital anatomical structure, there is a possibility of incomplete resection during surgery. We recommend that, in addition to the aforementioned WHO risk stratification, radiotherapy should be strongly considered when MRI or CT demonstrates tumor with bony invasion, or when intraoperative findings reveal dense adherence of the tumor to the extraocular rectus muscles or periosteum–findings that lead the surgeon to judge a high risk of residual disease and thus a high risk of recurrence. Previous literature has reported that for recurrent, metastatic, or malignant OSFT, especially cases with high risk of residual tumor or uncertain surgical margins, postoperative intensity-modulated radiotherapy (IMRT) significantly improves local control with good tolerance (24). This evidence strongly supports our argument for earlier implementation of adjuvant therapy in high-risk patient subgroups. However, large-scale clinical studies are needed to further validate this approach. Furthermore, preoperative neoadjuvant radiotherapy may represent a viable option for patients with multiple recurrences or those exhibiting highly aggressive biological behavior. These considerations regarding the timing of intervention and treatment strategy suggest that, beyond conventional surgical resection, individualized selection of radiotherapy timing for high-risk subgroups may contribute to improved outcomes.

Conventional chemotherapy, primarily anthracycline-based regimens, has shown low response rates in advanced SFT (25). No established effective chemotherapy exists for OSFT (6). To date, no dedicated clinical trials of targeted therapy specifically for OSFT have been conducted. Targeted therapy therefore remains in the exploratory stage and represents a promising avenue for future investigation. For solitary fibrous tumor, several targeted therapy strategies have been proposed in recent literature. FER kinase has been identified as a key therapeutic target through multi-omics analysis (26). Additionally, IDH1 mutations and PD-L1 overexpression have been detected in subsets of SFT patients, suggesting potential benefits of targeted therapy and immune checkpoint inhibition (27). Antiangiogenic agents remain the most established systemic treatment option for advanced SFT, with pazopanib demonstrating an objective response rate of 51% in a phase II trial (28). Emerging strategies, including RNA-targeting technologies (29) and others.

In view of the protracted clinical course, high recurrence rate, and metastatic potential of orbital solitary fibrous tumors, long-term follow-up should encompass regular imaging assessment of the primary site and extend to screening of common and critical metastatic locations, including the lungs (23) and brain. We therefore recommend that orbital and sinus MRIs be performed every 3–6 months during the first 5 years post-surgery, as studies have shown that approximately half of local recurrences and distant metastases of SFT occur within 5 years after surgery (30), followed by every 6–12 months thereafter for lifelong follow-up. Additionally, an annual chest CT is essential for lung metastasis screening, while biennial brain MRI should be conducted for brain metastasis surveillance. If distant metastasis is suspected, whole-body PET-CT is recommended for further evaluation to define the extent of metastatic disease.

## 4 Conclusion

Orbital solitary fibrous tumor is a rare yet potentially devastating mesenchymal neoplasm that, despite its generally indolent classification, can exhibit highly aggressive biological behavior characterized by intractable local recurrences and extensive adjacent tissue invasion. Its protracted development–as evidenced by the 18-years clinical course and five recurrences observed in our patient–highlights the substantial anatomical challenges of achieving complete R0 resection within the confined orbital cavity. The significant risk of irreversible structural destruction, visual impairment, and extensive sinonasal metastasis underscores the critical need of early detection and definitive management. To optimize disease control and improve patient outcomes, we advocate for a proactive and individualized therapeutic approach: prioritizing complete surgical excision as the primary modality, strategically integrating postoperative adjuvant radiotherapy for cases exhibiting aggressive histological features or bony wall destruction, and mandating lifelong, rigorous systemic and localized imaging surveillance to facilitate the timely detection of late recurrences and distant metastases.

## Data Availability

The raw data supporting the conclusions of this article will be made available by the authors, without undue reservation.
